# A Multiparametric MRI-Based Radiomics Nomogram for Preoperative Prediction of Survival Stratification in Glioblastoma Patients With Standard Treatment

**DOI:** 10.3389/fonc.2022.758622

**Published:** 2022-02-16

**Authors:** Xin Jia, Yixuan Zhai, Dixiang Song, Yiming Wang, Shuxin Wei, Fengdong Yang, Xinting Wei

**Affiliations:** Department of Neurosurgery, The First Affiliated Hospital of Zhengzhou University, Zhengzhou, China

**Keywords:** glioblastoma, preoperative survival stratification, machine learning, radiomics, nomogram

## Abstract

**Objective:**

To construct and validate a radiomics nomogram for preoperative prediction of survival stratification in glioblastoma (GBM) patients with standard treatment according to radiomics features extracted from multiparameter magnetic resonance imaging (MRI), which could facilitate clinical decision-making.

**Methods:**

A total of 125 eligible GBM patients (53 in the short and 72 in the long survival group, separated by an overall survival of 12 months) were randomly divided into a training cohort (n = 87) and a validation cohort (n = 38). Radiomics features were extracted from the MRI of each patient. The T-test and the least absolute shrinkage and selection operator algorithm (LASSO) were used for feature selection. Next, three feature classifier models were established based on the selected features and evaluated by the area under curve (AUC). A radiomics score (Radscore) was then constructed by these features for each patient. Combined with clinical features, a radiomics nomogram was constructed with independent risk factors selected by the logistic regression model. The performance of the nomogram was assessed by AUC, calibration, discrimination, and clinical usefulness.

**Results:**

There were 5,216 radiomics features extracted from each patient, and 5,060 of them were stable features judged by the intraclass correlation coefficients (ICCs). 21 features were included in the construction of the radiomics score. Of three feature classifier models, support vector machines (SVM) had the best classification effect. The radiomics nomogram was constructed in the training cohort and exhibited promising calibration and discrimination with AUCs of 0.877 and 0.919 in the training and validation cohorts, respectively. The favorable decision curve analysis (DCA) indicated the clinical usefulness of the radiomics nomogram.

**Conclusions:**

The presented radiomics nomogram, as a non-invasive tool, achieved satisfactory preoperative prediction of the individualized survival stratification of GBM patients.

## Introduction

Glioblastoma (GBM) is the most frequent primary malignant tumor of the central nervous system with the characteristics of highly aggressive growth, high recurrence rate, and poor prognosis, defined as Grade IV glioma or glioblastoma multiforme according to the 2016 World Health Organization classification of brain tumors (1). The current standard treatment includes maximal safe surgical resection followed by concomitant radiotherapy and adjuvant chemotherapy with temozolomide, then maintenance with 6–12 months of temozolomide as single-agent therapy (2, 3). Despite receiving standard treatment, the median overall survival (OS) of GBM patients remains 10–14 months (4, 5). Therefore, it is necessary to establish a more reasonable and personalized preoperative prediction approach for survival stratification so that neurosurgeons can make the evaluation, targeted treatment, follow-up management, and better education for GBM patients.

Recent investigative studies have identified several prognostic factors commonly used to predict the prognoses of GBM patients, such as the age of diagnosis, laterality, radiotherapy, chemotherapy (6), Karnofsky performance status (KPS), the extent of resection (total/gross, subtotal, or other), O6-methylguanine-DNA methyltransferase (MGMT) gene status (7), and isocitrate dehydrogenase (IDH) mutation status (8). Additionally, magnetic resonance imaging (MRI), as a non-invasive and non-radioactive inspection method, has indicated great potential in predicting the prognosis of GBM patients based on providing a comprehensive macro-image of the whole tumor (9, 10). Recently, as a potentially non-invasive high-throughput method of acquiring tumor characteristics, radiomics has been used in many tumors, including the pancreatic ductal adenocarcinomas, colorectal cancer, and pituitary adenomas (11–13).

In this research, the main purpose was to develop and then independently validate a nomogram based on radiomics score (Radscore) for preoperative prediction of individual OS stratification probabilities for GBM patients who would receive standard treatment. Moreover, three feature classifiers were established to evaluate the value of the selected radiomics features for differentiating survival stratification of GBM patients.

## Methods

### Patients

This retrospective study included 125 patients with newly diagnosed GBM undergoing open craniotomy at the First Affiliated Hospital of Zhengzhou University from January 2018 to January 2020 according to the following criteria. The inclusion criteria were as follows: 1) patients with GBM confirmed by pathological report; 2) patients with complete data of medical and imaging records before surgery; and 3) patients with standard treatment, i.e., maximal safe surgical resection followed by radiotherapy plus adjuvant chemotherapy with temozolomide, then maintenance with 6–12 months of temozolomide as single-agent therapy. The exclusion criteria were as follows: 1) patients with biopsy only; 2) patients without complete medical records; 3) patients with incomplete image data and image artifacts; and 4) patients with radiotherapy or chemotherapy alone after surgery. The medical ethics committee of the First Affiliated Hospital of Zhengzhou University approved this retrospective study.

There were 4 clinical features and 2 conventional imaging features collected for each patient, including age at diagnosis, gender, preoperative Karnofsky performance status (pKPS), preoperative epilepsy status (pEPI), located lobe (frontal, temporal, parietal, occipital, insular, corpus callosum), and hemisphere (left, right, bilateral). Regular follow-up was applied to every patient until death or June 2021 through the clinic or phone, once every month for the first 6 months after surgery, and every 3–6 months thereafter. Each patient was separated into short or long survival group according to the OS of 12 months. Then, the patients were divided randomly into training cohort (n = 87, 70%) used for model construction and validation cohort (n = 38, 30%) used for model evaluation.

### MRI Acquisition and Preprocessing

Four imaging sequences were selected from the head MRI undergone before surgery of each patient, i.e., T1-weighted contrast-enhanced imaging (T1C), T1-weighted imaging (T1), T2-weighted imaging (T2), and T2-weighted fluid-attenuated inversion recovery imaging (T2F). The imaging was performed on five models of MRI scanners from two manufacturers, i.e., Verio, Prisma, TrioTim, and Skyra of Siemens and Discovery MR750 of GE Medical Systems. The T1 sequence was acquired with the following range of parameters: repetition time (TR)/echo time (TE), 163–1,750.03 ms/2.46–25.176 ms; slice thickness, 5 mm; spacing between slices, 6.5–6.75 mm. The T1C sequence was acquired with the following range of parameters: TR/TE, 21–3,900 ms/2.32–92 ms; slice thickness, 0.9–5 mm; spacing between slices, 6.50–6.75 mm. The T2 sequence was acquired with the following range of parameters: TR/TE, 3,800–5,673.8213 ms/92–117 ms; slice thickness, 5 mm; spacing between slices, 6.5–6.75 mm. The T2F sequence was acquired with the following range of parameters: TR/TE, 5,000–8,400 ms/81–157.732 ms; slice thickness, 5 mm; spacing between slices, 6.5–6.75 mm.

Image preprocessing was performed by 3D Slicer software (v4.11.0). First, skull-stripping was executed by the Swiss Skull Stripper module, and T1, T2, and T2F sequence images were registered to T1C sequence images. Next, N4 bias field correction was applied to correct the intensity unevenness of each sequence. Ultimately, image normalization (normalizeScale = 100) and image resampling (ResamplePixelSpacing = [3, 3, 3]) were performed in Python environment by the PyRadiomics package.

### Tumor Delineation and Radiomics Feature Extraction

The region of interest (ROI) was delineated on T1C using 3D Slicer software separately by two neurosurgeons, who were blinded to the clinical data and had over 5 years of clinical experience.

The radiomics feature extraction was executed in the Python environment with the PyRadiomics package, which was an open-source python package for the extraction of Radiomics features from medical imaging. The **Supplementary Material Data S1** supplied the detail parameter settings of feature extraction. There were seven image types (Original, Wavelet, Laplacian of Gaussian (LoG sigma [3.0, 5.0]), Square, SquareRoot, Logarithm, Exponential) and six feature classes (shape, first-order statistics, gray-level co-occurrence matrix (glcm), gray-level run length matrix (glrlm), gray-level size zone matrix (glszm), gray-level dependence matrix (gldm)) adopted for each sequence.

### OS Status-Related Radiomics Feature Selection

Three feature selection steps were performed to avoid overfitting before establishing the model. First, the stable features, which were defined by intraclass correlation coefficients (ICCs) >0.8, were selected between the two groups of ROIs drawn by the two neurosurgeons (14). Next, the t-test was applied to each stable feature between short and long survival cases. Then, the least absolute shrinkage and selection operator (LASSO) regression algorithm was applied to analyze these features whose p-values were less than 0.05 in the t-test. The optimal λ value in LASSO was automatically selected by 10-fold cross-validation with a maximum area under the curve (AUC) criterion, where the final value of λ yielded the maximum AUC.

### Construction and Assessment of the Radiomics Nomogram

**Figure 1:**
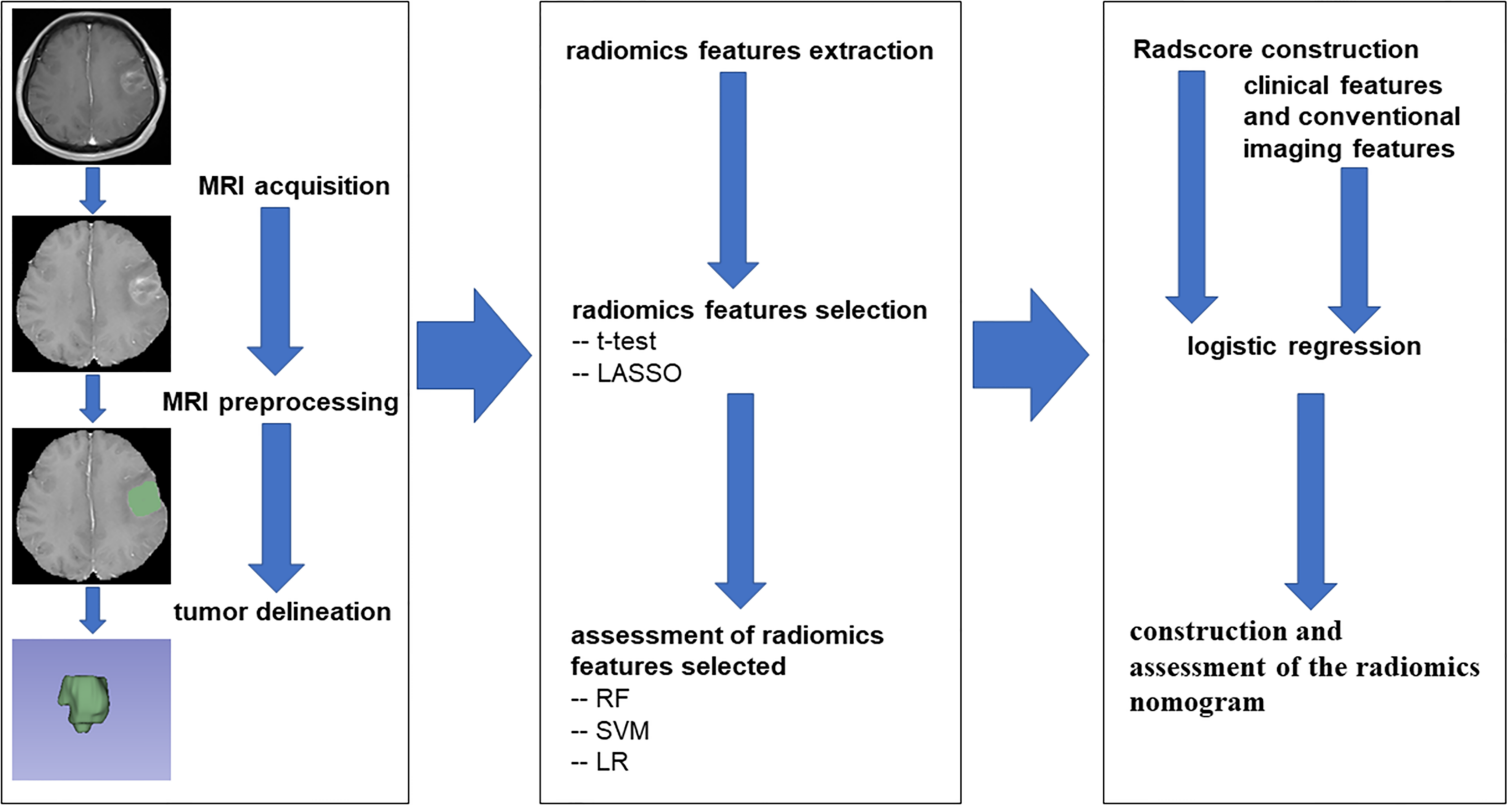
The flowchart of the study.

To understand the ability of these OS status-related radiomics features to discriminate the OS state, three supervised machine learning algorithms were applied in the training cohort, including random forest (RF) algorithm, support vector machine (SVM) algorithm, and logistic regression (LR) algorithm, and tested in the validation cohort. The performance of training and validation cohorts was evaluated by AUC. Then, these features with non-zero coefficients were used to construct the Radscore for each patient. Finally, the radiomics nomogram based on Radscore from the training cohort was established by logistic regression and assessed by the validation cohort. For the two cohorts, the discriminative ability of the radiomics nomogram was quantitatively measured using AUC, and the calibration curves were plotted based on observed probabilities and the nomogram-estimated probabilities (15). To evaluate the clinical utility of the radiomics nomogram, the decision curve analysis (DCA) was executed by calculating the net benefits at different threshold probabilities in the combined training and validation cohorts (16). The flowchart of this research is shown in **Figure 1**.

### Statistical Analysis

The continuous variables were analyzed by Student’s t-test or Mann–Whitney U-test, and chi-square test, Yates’ correction, or Fisher’s exact probabilities were performed in the categorical characteristics. Generally, a two-sided p-value < 0.05 was considered statistically significant. All data analyses were performed in Python (v3.7.6) and R software (v4.0.5).

## Results

### Patient Summary

A total of 320 newly diagnosed GBM patients were collected. According to the inclusion and exclusion criteria, we included 177 patients whose image quality met the criteria and 52 patients without standard treatment were excluded. 125 patients were finally included in our study (87 cases in the training cohort and 38 cases in the validation cohort). The clinical features, conventional imaging features, and Radscores of patients in the training cohort, validation cohort and total cohort, are summarized in **Table 1**. There were no significant differences between the short OS and long OS groups in age, gender, pEPI, pKPS, located lobe (frontal, temporal, parietal, occipital, insular, corpus callosum), and hemisphere (left, right, bilateral). However, it was worth noting that Radscore had significant differences in the short OS and long OS groups (p < 0.001).

**Table 1:** Characteristics of GBM patients in the training cohort and validation cohort.

Variable	Training cohort (n = 87)			Validation cohort (n = 38)				Total cohort (n = 125)		
	Short OS	Long OS	p-value	Short OS		Long OS	p-value	Short OS	Long OS	p-value
Age/year (mean ± SD)	56.70 ± 14.70	52.82 ± 10.58	0.207		50.17 ± 17.36	50.53 ± 12.96	0.946	53.87 ± 16.08	52.35 ± 11.06	0.555
Gender			0.571				0.945			0.540
Male	16	34			12	8		28	42	
Female	14	23			11	7		25	30	
pEPI			0.624				0.143			0.199
Yes	5	12			2	4		7	16	
No	25	45			21	11		46	56	
pKPS	74.33 ± 14.31	72.28 ± 15.59	0.550		74.35 ± 12.73	74.00 ± 12.42	0.934	74.34 ± 13.52	72.64 ± 14.92	0.514
Located lobe										
Frontal	14	17	0.158		7	5	1.000	21	22	0.292
Temporal	7	17	0.618		4	7	0.073	11	24	0.122
Parietal	1	11	0.084		5	2	0.681	6	13	0.300
Occipital	3	2	0.452		1	0	1.000	4	2	0.418
Insular	5	6	0.502		4	1	0.630	9	7	0.230
Corpus callosum	0	4	0.344		2	0	0.510	2	4	0.970
Hemisphere										
Left	16	32	0.802		11	7	0.793	27	39	0.721
Right	13	21	0.555		10	8	0.793	23	29	0.727
Bilateral	1	4	0.828		2	0	0.667	3	4	0.713
Radscore										
Mean	-0.167	0.128	<0.001		-0.207	0.165	<0.001	-0.185	-0.136	<0.001
Range	(-0.411,0.182)	(-0.405,0.609)			(-0.528,0.150)	(-0.057,0.613)		(-0.528,0.182)	(-0.405,0.613)	
Median OS/month	16		NA	11.5			NA	15		NA

SD, standard deviation; NA, not applicable.

### Radiomics Feature Analysis and Radscore Calculation

**Figure 2:**
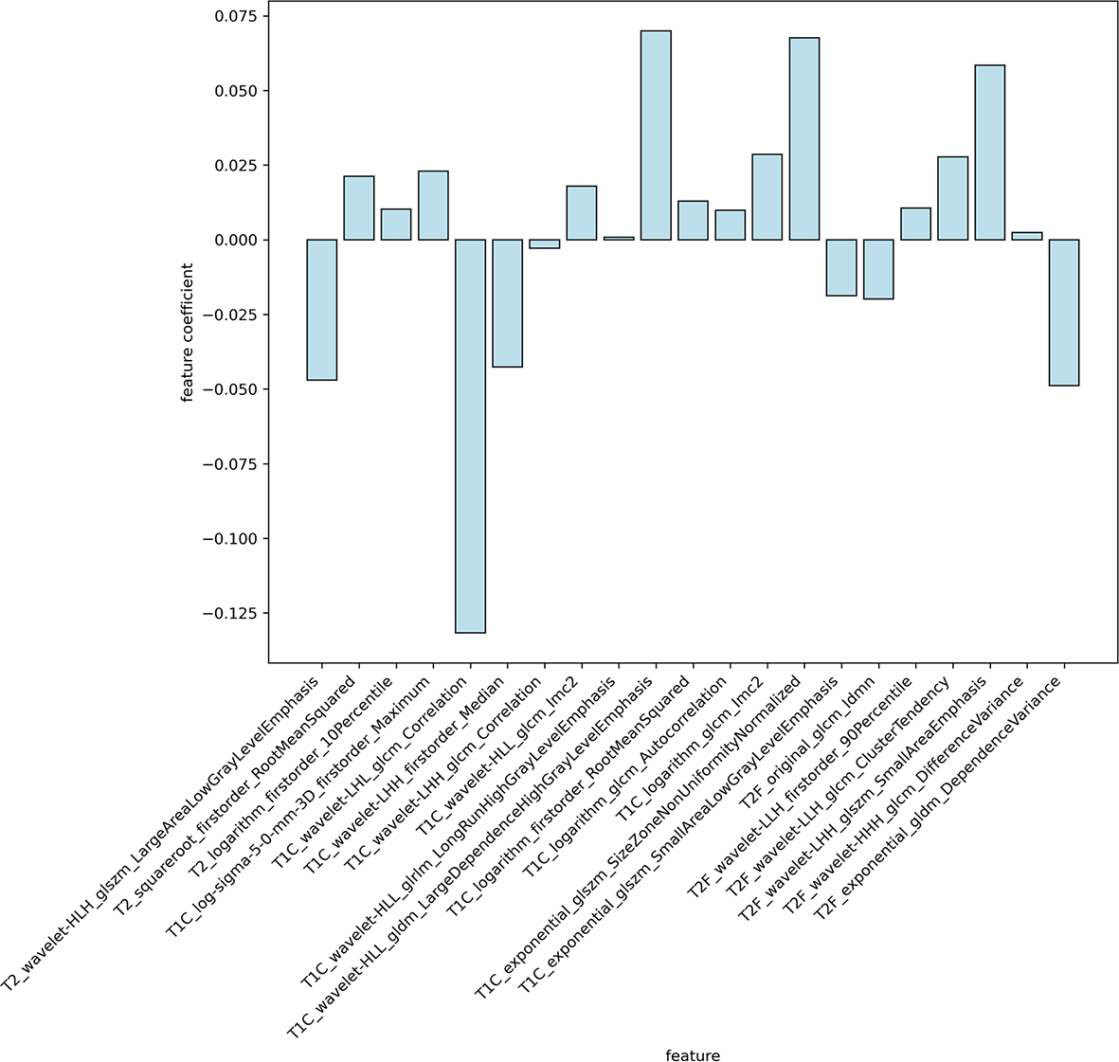
The weights of radiomics features selected. It could be seen that the T1C sequence had a greater impact on OS stratification.

In this study, a total of 1,304 radiomics features for each sequence were extracted, including 14 shape features, 18 first-order statistics features, 68 texture features, 172 LoG features, 688 wavelet features, 86 square features, 86 square-root features, 86 logarithm features, and 86 exponential features. A total of 5,216 radiomics features were calculated from four imaging sequences for each patient. Among them, 5,080 radiomics features were stable after being screened by ICCs. After that, 777 radiomics features were selected by t-test. Ultimately, the optimal regulation weight λ (λ = 0.029470517025518096) was determined for the LASSO algorithm, and 21 features with non-zero coefficients were selected for OS stratification of GBM patients. The detailed names and weights of the 25 radiomics features are shown in **Table 2** and **Figure 2**. It could be seen that the T1C sequence had a greater impact on OS stratification.

**Table 2:** Description of the radiomics features selected.

Sequence	Image type	Feature class	Feature name
T2	HLH wavelet	glszm	LargeAreaLowGrayLevelEmphasis
T2	SquareRoot	firstorder	RootMeanSquared
T2	Logarithm	firstorder	10Percentile
T1C	log(sigma=5.0mm)	firstorder	Maximum
T1C	LHL wavelet	glcm	Correlation
T1C	LHH wavelet	firstorder	Median
T1C	LHH wavelet	glcm	Correlation
T1C	HLL wavelet	glcm	lmc2
T1C	HLL wavelet	glrlm	LongRunHighGrayLevelEmphasis
T1C	HLL wavelet	gldm	LargeDependenceHighGrayLevelEmphasis
T1C	Logarithm	firstorder	RootMeanSquared
T1C	Logarithm	glcm	Autocorrelation
T1C	Logarithm	glcm	lmc2
T1C	Exponential	glszm	SizeZoneNonUniformityNormalized
T1C	Exponential	glszm	SmallAreaLowGrayLevelEmphasis
T2F	Original	glcm	Idmn
T2F	LLH wavelet	firstorder	90Percentile
T2F	LLH wavelet	glcm	ClusterTendency
T2F	LHH wavelet	glszm	SmallAreaEmphasis
T2F	HHH wavelet	glcm	DifferenceVariance
T2F	Exponential	gldm	DependenceVariance

There were three supervised machine learning algorithms models constructed to determine the ability of these OS status-related radiomics features to discriminate the OS stratification. The detail performances of the three models are shown in **Table 3**. The SVM model performed best among the three models. The AUC, sensitivity, accuracy, and F1 score were 0.75, 0.93, 0.71, and 0.72 in the validation cohort, respectively.

**Table 3:** Comparison of the three radiomics feature classifiers.

Variable	RF		SVM		LR	
	Training	Validation	Training	Validation	Training	Validation
AUC	0.98	0.72	0.97	0.75	0.85	0.73
Sensitivity	0.98	0.87	1	0.93	0.84	0.8
Specificity	0.98	0.57	0.95	0.57	0.86	0.65
Accuracy	0.98	0.68	0.97	0.71	0.85	0.71
F1-score	0.98	0.68	0.97	0.72	0.85	0.69

RF, random forest; SVM, support vector machine; LR, logistic regression.

**Figure 3:**
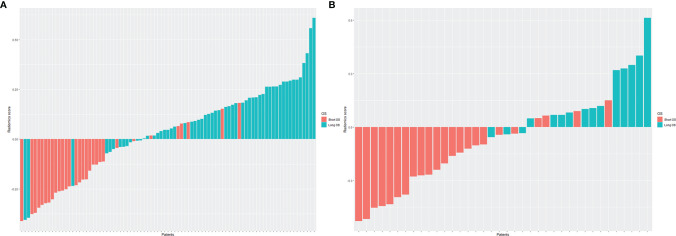
The histogram of Radscore for each patient in the training cohort **(A)** and validation cohort **(B)**. The red bars showed the Radscore values for the short OS patients, and the blue bars showed the values for the long OS patients. Patients with long OS showed higher Radscores than patients with short OS in both the training and validation cohorts.

Then, the Radscore for each patient in training and validation cohorts was constructed for further analysis, which was calculated by multiplying each feature coefficient by the corresponding feature value and summing. The corresponding fitting formula is listed in **Supplementary Material Data S2**. Patients with long OS showed higher Radscores than patients with short OS in both the training and validation cohorts **(** **Figure 3****)**. In the training cohort, the average values of Radscore were significant differences in the short OS and long OS groups (-0.167 vs. 0.128, p < 0.001). Similarly, the mean Radscore of long OS was 0.165, which was significantly higher than that of short OS (-0.207, p < 0.001) in the validation cohort.

### Radiomics Nomogram Establishment and Evaluation

To establish the radiomics nomogram, the logistic regression based on Radscore, clinical features, and conventional imaging features was applied to find independent predictors of OS stratification by univariate and multivariable logistic regression. The results of logistic regression are presented in **Table 4**, which demonstrated that only Radscore was the significant independent predictor for OS stratification.

**Table 4:** The results of logistic regression.

Variable	Univariate logistic regression		Multivariable logistic regression	
	OR (95% CI)	p-value	OR (95% CI)	p-value
Age	0.972 (0.930–1.010)	0.164	NA	NA
Gender	1.293 (0.528–3.167)	0.571	NA	NA
pEPI	1.333 (0.439–4.585)	0.625	NA	NA
pKPS	0.991 (0.960–1.020)	0.545	NA	NA
Frontal	0.486 (0.193–1.213)	0.122	NA	NA
Temporal	1.396 (0.518–4.067)	0.521	NA	NA
Parietal	6.935 (1.249–130.091)	0.070	NA	NA
Occipital	0.327 (0.041–2.085)	0.236	NA	NA
Insular	0.588 (0.162–2.216)	0.416	NA	NA
Corpus callosum	0.452 (0.012–3.245)	0.980	NA	NA
Left	1.120 (0.458–2.729)	0.802	NA	NA
Right	0.763 (0.309–1.893)	0.556	NA	NA
Bilateral	2.189 (0.306–43.891)	0.493	NA	NA
Radscore	5941.499 (239.983–336406.47)	<0.001	5941.499 (239.983–336406.47)	<0.001

OR, odd ratio; CI, confidence interval; NA, not applicable.

**Figure 4:**
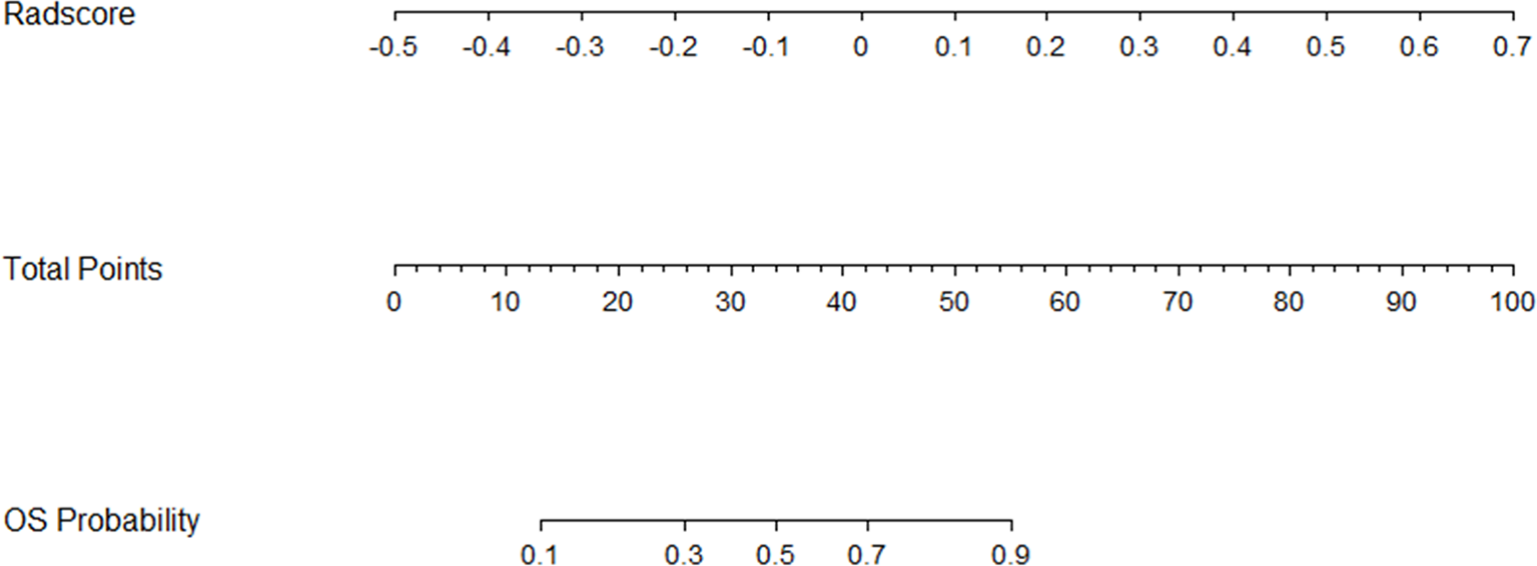
The radiomics nomogram for OS stratification of GBM patients.

**Figure 5:**
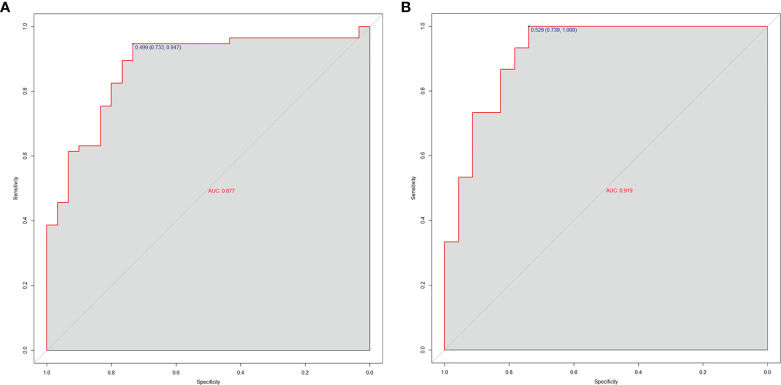
The AUCs of the radiomics nomogram for the training cohort **(A)** and validation cohort **(B)**. The results demonstrated that the radiomics nomogram performed well in both groups with favorable sensitivity and specificity.

**Figure 6:**
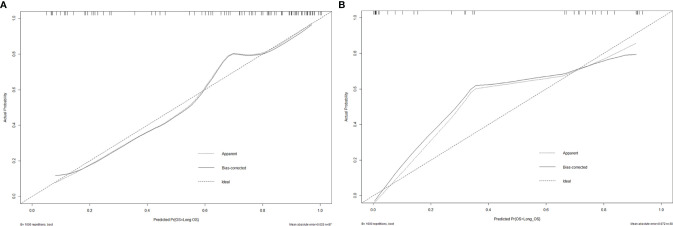
The calibration curves of the radiomics nomogram for the training cohort **(A)** and validation cohort **(B)**. It showed the agreement between observed probabilities and the nomogram-estimated probabilities.

**Figure 7:**
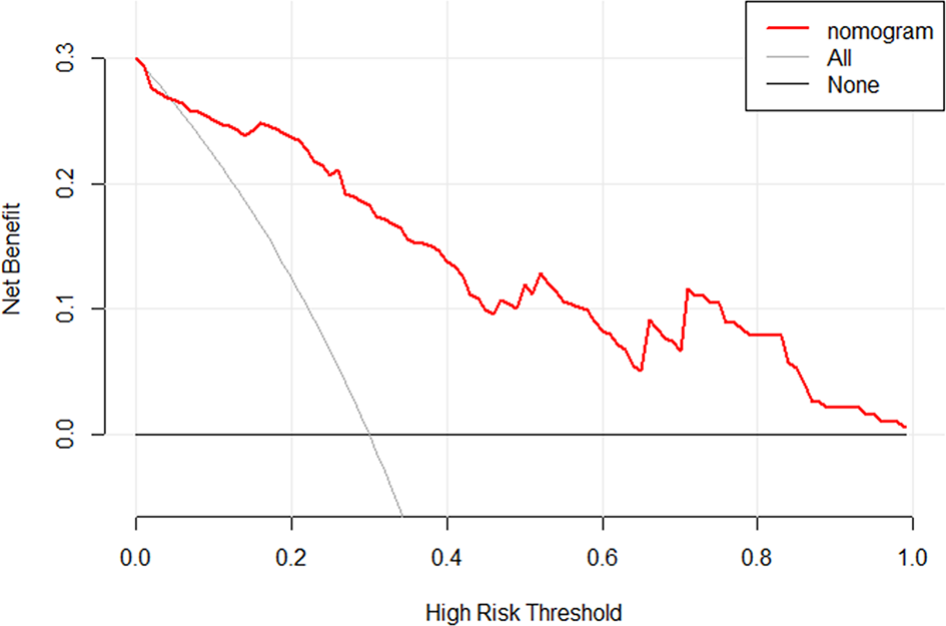
The DCA for the developed radiomics nomogram. The y-axis represents the net benefit. The x-axis represents the threshold probability. The black line at the bottom named “None” represented the hypothesis that no patients had long OS, which meant the net benefit would be zero if all patients did not have the long OS. The gray line named “All” represents the hypothesis that all patients had long OS. The red line represents the net benefit of the radiomics nomogram at different threshold probabilities. The result indicated the radiomics nomogram to stratify the OS of GBM patients could yield clinical net benefits.

Then, the radiomics nomogram was constructed according to the multivariable logistic regression **(** **Figure 4****)**. The ROC curve, which was based on the probability of long OS according to the Radscore, was used to evaluate the sensitivity and specificity of the nomogram. The AUCs of the nomogram were 0.877 and 0.919 in the training and validation cohorts, respectively, which indicated favorable sensitivity and specificity **(** **Figure 5****)**. The calibration curve of the proposed nomogram based on the training cohort was constructed, and a favorable calibration was confirmed in the validation cohort **(** **Figure 6****)**. Moreover, the result of DCA showed that the nomogram to stratify the OS of GBM patients could yield clinical net benefits **(** **Figure 7****)**.

## Discussion

A total of 125 newly diagnosed GBM patients with standard treatment were included in this study, and the objective was to develop and validate a preoperative prediction model for OS stratification. Only one independent predictive factor was found to be associated with OS stratification. The Radscore factor was adopted in a clinically relevant nomogram model that can predict the probabilities of OS stratification for the GBM patients. The radiomics nomogram demonstrated that a patient was more likely to have long OS if he had a higher Radscore before operation and would receive standard treatment. This may help neurosurgeons with preoperative planning and allow for better education for these patients or those family members who were extremely concerned about the postoperative survival or hesitated to continue the treatment for various reasons before the operation.

The nomogram, as a tool of prediction, integrates a variety of independent predictive factors and visualizes the overall impact of these factors on survival in each patient to help clinicians develop intervention plans (17). Owing to its convenience and accuracy, the nomogram has been used in many tumors, such as pancreatic ductal adenocarcinomas, colorectal cancer, pituitary adenomas, and gastric cancer (11–13, 18). Moreover, radiomics, as a new study method, extracts, processes, and analyzes the quantitative and high-throughput data from medical imaging to explore their relationships with valuable information. When combined with radiomics features and traditional clinical features to construct a nomogram, the radiomics features showed stronger robustness, which had been confirmed by some studies (9, 19).

At present, there have been some studies that make efforts to predict the OS of GBM. These studies have selected some independent clinical risk factors related to OS, such as age at diagnosis, gender, KPS, MGMT, IDH, radiotherapy, chemotherapy, and radiotherapy combined with chemotherapy (8, 20, 21). In our research, the study population was newly diagnosed GBM patients who had undergone standard treatment, so no treatment factors were included. The factors included in our study were clinical features and conventional imaging features, and the postoperation-related factors were excluded including completeness of resection, pathological features, and treatment, for the study stage was defined as preoperation. Ultimately, according to the logistic regression, none of these clinical factors and conventional imaging features was selected as an independent risk factor. This result was different from the results of these studies (8, 20, 21). For this difference, we think it was because of strict treatment and imaging standards. On the other hand, it also indicated that patients who chose standard treatment after surgery were relatively concentrated in this study.

Recently, several studies about the nomogram for predicting survival of GBM based on radiomics were published. Zhang et al. (9) developed a radiomics nomogram, which showed excellent performance with 0.974 of the concordance index (C-index) in survival stratification. The C-index represents the AUC of ROC that plots sensitivity against 1-specificity of the radiomics nomogram (15). A total of 4,000 radiomics features were extracted from multiple regions of the GBM using multiparametric MRI, and 25 selected features were used for constructing the Radscore. Among these features, the T1C and T2F sequence of GBM contributed more than other MRI sequences. Xu et al. (22) reported a radiomics nomogram integrated with Radscore, ependymal, and pia mater involvement and age at diagnosis to stratify the survival of GBM patients, and the ROC reached up to 0.858. In this study, the data from Brain Tumor Segmentation Challenge 2018 were divided into training and test sets to build the model, and the data from the local medical center were used to validate the model. In our study, we first analyzed the extracted radiomics features with three machine learning algorithms to determine the ability of these OS status-related radiomics features to discriminate the OS stratification. The results showed that the three classifiers were all excellent, and the SVM performance was best among them (the AUCs of 0.97 and 0.75 in the training and validation cohorts), which illustrated the favorable ability for these features to stratify the OS of GBM patients. Similarly to the published researches, the nomogram only including Radscore represented the favorable ability to predict the long OS patients with 0.919 of the AUC in the validation set and these features from the T1C sequence had a greater impact on OS stratification, although we merely cared about preoperative features to stratify the OS of GBM patients.

However, our study still has some limitations. Firstly, this was not a multicenter study, although we had independently validated the model. More datasets from multimedical centers are needed to independently validate the robustness and repeatability of the radiomics nomogram. Second, although with high efficiency and sparsity, the combination method of the t-test and LASSO regression may be less stable when a large number of features were involved in the model. Other feature selection methods should be investigated in future work. Finally, these MRI images come from different imaging scanners and models, which may cause heterogeneity bias. In order to avoid this situation, all MRI images involved in the study were normalized and resampled before feature extraction. The same scanner and model are expected for MRI images in future researches for the convenience of image processing.

In conclusion, to help neurosurgeons make better preoperative planning and patient education, our research developed and validated a radiomics nomogram based on multiparameter MRI imaging. The presented radiomics nomogram, as a noninvasive tool, achieved satisfactory preoperative prediction of the individualized survival stratification of GBM patients.

## Data Availability Statement

The raw data supporting the conclusions of this article will be made available by the authors, without undue reservation.

## Ethics Statement

The studies involving human participants were reviewed and approved by the Ethics Committee of the First Affiliated Hospital of Zhengzhou University. Written informed consent for participation was not required for this study in accordance with the national legislation and the institutional requirements.

## Author Contributions

XJ and XW designed the study. XJ, DS, YW, and SW collected the clinical and radiomics data. XJ and FY preprocessed patients’ MR imaging and drew the ROI. XJ, DS, and YZ analyzed the data and developed the prediction model. XJ wrote the manuscript. All authors contributed to the article and approved the submitted version.

## Conflict of Interest

The authors declare that the research was conducted in the absence of any commercial or financial relationships that could be construed as a potential conflict of interest.

## Publisher’s Note

All claims expressed in this article are solely those of the authors and do not necessarily represent those of their affiliated organizations, or those of the publisher, the editors and the reviewers. Any product that may be evaluated in this article, or claim that may be made by its manufacturer, is not guaranteed or endorsed by the publisher.
