# Supplementary material for: A Multiparametric MRI-Based Radiomics Nomogram for Preoperative Prediction of Survival Stratification in Glioblastoma Patients With Standard Treatment
Source: Front Oncol. 2022 Feb 16;12:758622. doi: 10.3389/fonc.2022.758622 (PMC8888684; doi:10.3389/fonc.2022.758622)
Supplement: Supplementary file 1 [file DataSheet_1.docx]

**Data S1: The detail parameter settings of feature extraction**

imageType:

Original: {}

LoG:

sigma: [3.0, 5.0]

Wavelet: {}

Square: {}

SquareRoot: {}

Logarithm: {}

Exponential: {}

featureClass:

shape:

firstorder:

glcm:

- 'Autocorrelation'

- 'JointAverage'

- 'ClusterProminence'

- 'ClusterShade'

- 'ClusterTendency'

- 'Contrast'

- 'Correlation'

- 'DifferenceAverage'

- 'DifferenceEntropy'

- 'DifferenceVariance'

- 'JointEnergy'

- 'JointEntropy'

- 'Imc1'

- 'Imc2'

- 'Idm'

- 'Idmn'

- 'Id'

- 'Idn'

- 'InverseVariance'

- 'MaximumProbability'

- 'SumEntropy'

- 'SumSquares'

glrlm:

glszm:

gldm:

setting:

normalize: True

normalizeScale: 100

interpolator: 'sitkBSpline'

resampledPixelSpacing: [3, 3, 3]

binWidth: 5

voxelArrayShift: 300

label: 1

**Data S2: The fitting formula of Radscore**

Radscore =

(-0.046967) * [T2_wavelet-HLH_glszm_LargeAreaLowGrayLevelEmphasis]

+ (0.021273) * [T2_squareroot_firstorder_RootMeanSquared]

+ (0.010179) * [T2_logarithm_firstorder_10Percentile]

+ (0.022998) * [T1c_log-sigma-5-0-mm-3D_firstorder_Maximum]

+ (-0.131650) * [T1c_wavelet-LHL_glcm_Correlation]

+ (-0.042666) * [T1c_wavelet-LHH_firstorder_Median]

+ (-0.002805) * [T1c_wavelet-LHH_glcm_Correlation]

+ (0.017896) * [T1c_wavelet-HLL_glcm_Imc2]

+ (0.000792) * [T1c_wavelet-HLL_glrlm_LongRunHighGrayLevelEmphasis]

+(0.069866) *[T1c_wavelet-HLL_gldm_LargeDependenceHighGrayLevelEmphasis]

+ (0.012945) * [T1c_logarithm_firstorder_RootMeanSquared]

+ (0.009902) * [T1c_logarithm_glcm_Autocorrelation]

+ (0.028593) * [T1c_logarithm_glcm_Imc2]

+ (0.067637) * [T1c_exponential_glszm_SizeZoneNonUniformityNormalized]

+ (-0.018813) * [T1c_exponential_glszm_SmallAreaLowGrayLevelEmphasis]

+ (-0.019815) * [T2f_original_glcm_Idmn]

+ (0.010603) * [T2f_wavelet-LLH_firstorder_90Percentile]

+ (0.027761) * [T2f_wavelet-LLH_glcm_ClusterTendency]

+ (0.058428) * [T2f_wavelet-LHH_glszm_SmallAreaEmphasis]

+ (0.002383) * [T2f_wavelet-HHH_glcm_DifferenceVariance]

+ (-0.048829) * [T2f_exponential_gldm_DependenceVariance]
